# Differential Pasting and Rheological Properties of Diverse Underutilized Starches Modified by Acetic Anhydride and Vinyl Acetate

**DOI:** 10.3390/foods14132227

**Published:** 2025-06-24

**Authors:** Song Xu, Bilatu Agza Gebre, Chuangchuang Zhang, Solomon Abate Mekonnen, Mengting Ma, Hui Zhang, Zhongquan Sui, Harold Corke

**Affiliations:** 1 College of Food Science and Engineering, Shandong Agricultural University, Tai’an 271018, China; 2023110540@sdau.edu.cn; 2 Department of Food Science & Technology, School of Agriculture and Biology, Shanghai Jiao Tong University, Shanghai 200240, China; bilatuagza@gmail.com (B.A.G.); cczhang@sjtu.edu.cn (C.Z.); zsui@sjtu.edu.cn (Z.S.); 3 Department of Food Science & Nutrition, Ethiopian Institute of Agricultural Research, Addis Ababa 999047, Ethiopia; solomon.abt@gmail.com; 4 Department of Food Science and Nutrition, Culinary Institute, University of Jinan, Jinan 250022, China; 5 Biotechnology and Food Engineering Program, Guangdong Technion-Israel Institute of Technology, Shantou 515063, China; harold.corke@gtiit.edu.cn; 6 Faculty of Biotechnology and Food Engineering, Technion-Israel Institute of Technology, Haifa 3200003, Israel

**Keywords:** underutilized starches, acetylation, amylose, viscosity

## Abstract

Underutilized starch sources are gaining increasing recognition. However, the inherent functional deficiencies of native starch have limited its application in food industry. To counteract the deficiencies in its native characteristics, starch can be modified by acetylation. Two waxy starches (proso millet and amaranth) and four non-waxy starches (foxtail millet, quinoa, buckwheat, and oat) were modified by acetic anhydride and vinyl acetate, respectively. Degree of substitution of acetylated starches revealed that granule size did not significantly affect acetylation efficiency in starches from different plant origins. Acetylation increased peak and final viscosity of starches, with vinyl acetate exhibiting a more pronounced effect than acetic anhydride. Acetic anhydride decreased K and increased n values of non-waxy starches, showing reduced thickening ability. In contrast, vinyl acetate modification showed opposite trends, suggesting increased viscosity and pseudoplasticity. For non-waxy starches, G′_25°C_, G′_0.1Hz_, G′_20Hz_ and gel hardness decreased after acetylation, indicating that acetylation contributed to a less solid and less elastic gel network. The extent of change in vinyl acetate modification was more pronounced than that of acetic anhydride. For waxy starch, vinyl acetate modification decreased tan δ_25°C_ and increased gel hardness. In summary, acetylation reagent type was the major factor determining the pasting properties of acetylated starch, but the presence or absence of amylose would influence the rheological and gel properties of acetic anhydride and vinyl acetate modified starches. These findings could help unlock the potential applications of acetylated underutilized starches in the food industry.

## 1. Introduction

Underutilized starch source, such as foxtail millet, proso millet, buckwheat, quinoa, oats, and amaranth, are gaining increasing recognition due to their remarkable health benefits. Proso (*Panicum miliaceum*) and foxtail millets (*Setaria italica*) are the oldest cultivated millet crops and are often grown in harsh conditions because of their better adaptability to abiotic stress than most other crops [1]. Common buckwheat (*Fagopyrum esculentum* Moench.) is used as a functional food because of its high content of flavonoids [2]. Oat (*Avena sativa*), is widely consumed as whole-grain foods due to their ability to lower cholesterol and assist in managing diabetes. Quinoa (*Chenopodium quinoa* Willd) and amaranth (*Amaranthus* sp.) belong to the Chenopodiaceae family, and they are recognized for their nutritional significance [3]. Although numerous studies have been conducted to uncover the potential health benefits of bioactive components found in such crops, the starch that remains after the extraction of these components has been underutilized. Further, investigation of these starches is crucial to encourage the consumption of such underutilized materials and to explore their possible applications. However, the inherent functional deficiencies of native starch, such as poor solubility and dispersibility in cold water, thermal instability during gelatinization and retrogradation, and sensitivity to shear during processing, have limited its application in food industry.

To enhance the desired techno-functionalities and counteract the deficiencies in its native characteristics, starch properties are often enhanced by chemical modifications [4]. Acetylation is a common chemical modification, with acetylated starch of low degree of substitution (DS < 0.1) being used for various food applications. Acetylated starch has a lower gelatinization temperature, higher gelatinization transparency, higher solubility and swelling capacity, higher viscosity, and higher thermal stability compared to native starch [4]. Starch pastes consist of fragments of swollen granules, aggregates of swollen starch and dissolved starch molecules. The characteristics of acetylated starch pastes, such as their viscosity, transparency, shear resistance and retrogradation tendency, are key factors determining starch applications in the food industry [5]. Rheological properties, including viscosity, elasticity, and viscoelastic behavior, are critical in determining the texture, stability, and overall quality of starch-based products [5]. Understanding the rheological behavior of acetylated starch pastes is essential for optimizing their performance in various food applications, such as thickening, gelling, and stabilizing agents.

The pasting and rheological properties of acetylated starch depend on various factors including botanical source, acetylation reagents, degree of substitution and granule structure [6]. Acetylation involves the esterification of starch by acetic anhydride or vinyl acetate in the presence of an alkaline catalyst. Acetic anhydride and vinyl acetate are reagents of different nature. Huang et al. found that acetic anhydride is a fast acetylation reagent, while vinyl acetate is a slow acetylation reagent [7,8]. Moreover, vinyl acetate starch exhibits higher granule swelling and peak viscosity than acetic anhydride starch at similar DS level [7]. Huang et al. concluded that the reason for this phenomenon was that acetyl groups of acetic anhydride modification are more intensely concentrated on the granule surfaces, whereas in vinyl acetate modified granules, they are more uniformly distributed throughout the granule [7]. However, our previous study found that the distribution of acetyl groups in starch granule was affected by the presence or absence of amylose; meanwhile, vinyl acetate was more effective in changing the starch supramolecular structure than was acetic anhydride [9]. Therefore, the acetylation reagents and the presence of amylose may affect the distribution of acetyl groups, thereby influencing the pasting and rheological properties of acetylated starch. Underutilized starches differ in amylose and amylopectin content, potentially providing a wider range of properties after acetylation. Despite these promising characteristics, there has been no systematic investigation into the effect of reagent type on the pasting and rheological properties of acetylated starch from these diverse crops.

In this study, underutilized starches were selected, including waxy proso millet (PMS) and amaranth (AS) starches, non-waxy foxtail millet (FMS), quinoa (QS), buckwheat (BS), and oat (OS) starches, generally have small granules (size < 10 µm) [10], which may have good reaction efficiency on modification due to the larger specific surface area of smaller granules. These underutilized starches were acetylated with acetic anhydride and vinyl acetate to systematically investigate the effects of reagent type on the pasting and rheological properties. The acetylated starches were prepared with a low degree of substitution (DS < 0.1). The changes in properties were compared to two reference acetylated cereal starches (waxy and normal maize). We hypothesized that acetylation by these two reagents would differently affect the properties of waxy and non-waxy starch. The findings from this study are expected to expand the potential applications of underutilized starches in the food industry.

## 2. Materials and Methods

### 2.1. Materials

Proso millet, amaranth, oat, buckwheat, quinoa, foxtail millet, and grains were purchased from a local market. Waxy maize starch (WMS) and normal maize starch (NMS) were purchased from Gaofeng Technology Co. (Suzhou, China). Acetic anhydride and vinyl acetate were purchased from Sigma-Aldrich (St. Louis, MO, USA). All other chemicals used in this study were of analytical grade and were obtained from Aladdin Reagent Co., Ltd. (Shanghai, China).

### 2.2. Starch Extraction

Starches were obtained following a previous reported method [11]. The grains were soaked in 1 L of NaOH (4 g/L) and left at 4 °C overnight, after which they were washed to remove the alkaline solution. The grains were then crushed and passed through a 200-mesh sieve. The filtrate was centrifuged to remove the supernatant and protein layer. This washing process was repeated until the starch suspension became neutral and the protein was completely removed. Finally, the starch was suspended in ethanol, subjected to filtration for 48 h, dried, and ground through a 100-mesh sieve to obtain starch. Amylose content of starch was determined using a colorimetric amylose-I_2_ complex formation following the previous method [12]. Abbreviation and Amylose contents of native starches are shown in Table 1.

### 2.3. Preparation of Acetic Anhydride Modified Starch

Acetic anhydride modified starch was prepared according to the method of Huang et al. [7], with minor modification. Briefly, starches (33.33 g, db.) were weighed and mixed with 75 g distilled water in a beaker, the suspension was stirred at 500 rpm for 15 min, then its pH adjusted to 8.4 by 3% NaOH. Acetic anhydride reagent (1.68 g) was slowly added to the starch suspension under a pH controlled to 8.0–8.4 using 3% NaOH. After 1 h, the reaction was stopped with 0.5 M HCl to adjust to pH 6.5. The starch suspension was centrifuged at 2000× *g* for 15 min and washed with distilled water three times. Finally, samples were mixed with ethanol and filtered through a Büchner funnel, then air-dried in a fume hood for 48 h.

### 2.4. Preparation of Vinyl Acetate Modified Starch

Vinyl acetate modified starch was prepared following the method of Huang et al. [7], with minor modification. Briefly, 75 g distilled water mixed with 3.27 g sodium carbonate and ~1 mL concentrated sulfuric acid was slowly added to ensure pH 10. Starches (33.33 g, db.) were weighed and mixed with 75 mL of buffer in a beaker, the suspension was stirred at 500 rpm for 15 min, then 1.417 g vinyl acetate reagent was directly added to the starch suspension. After 1 h, the reaction was stopped with 0.5 M HCl by adjusting to pH 6.5. The starch suspension was centrifuged at 2000× *g* for 15 min and washed with distilled water three times. Finally, samples were mixed with ethanol and filtered through a Büchner funnel, then air-dried in a fume hood for 48 h.

### 2.5. Degree of Substitution (DS) Determination

DS was determined using the titration method according to the previous method [9]. Starch (5 g) was mixed with 50 mL water and 3 drops of 10 g/L phenolphthalein indicator, and then the starch suspension was titrated by 0.1 M NaOH solution until slightly red (pH 8.6). Starch suspension was added to 25 mL 0.45 M NaOH solution and saponified in 50 °C water bath for 2 h. The suspension was neutralized with 0. 2 M HCl standard solution to the initial state (slightly red, pH 8.6), and the volume of the standard HCl solution used for titration was recorded as V_1_ (mL). For unmodified starch samples, the volume V_0_ (mL) of the standard solution of HCl used for titration was recorded using the same determination method as above. DS_TT_ was calculated following Equation (1):

(1) $$DS=\frac{0.162\times (V0-V1)\times 0.2}{1-0.043\times \left(V0-V1\right)\times 0.2}$$

where 0.162 corresponds to the molecular weight of the anhydroglucose unit (162 g/mol) divided by 1000 for unit conversion (g/mmol), 0.043 represents the molecular weight of the acetyl group (CH_3_CO^−^, 43 g/mol) divided by 1000 (43 g/mol ÷ 1000 = 0.043 g/mmol), and 0.2 is the molarity of standardized HCl solution used for titration (0.2 mol/L).

Abbreviation of acetic anhydride and vinyl acetate modified starches and DS values of samples are shown in Table 1.

### 2.6. Particle Size Distribution 

Particle size distributions of six underutilized starches and two reference maize starches before and after acetylation was determined by a laser particle analyzer (Mastersizer 3000, Malvern Instruments Ltd., Malvern, UK). A refractive index of 1.33 was used to calculate the particle size of starch. The 10th (d10), medium (d50), 90th (d90) percentile, and span factors of the granules were recorded.

### 2.7. Determination of Pasting Properties 

The pasting properties of the starch samples were determined using a Rapid Visco-Analyser (RVA 4500, Perten Instruments, Hägersten, Sweden). Starch samples (1.96 g, dry basis) were mixed with distilled water to reach a total weight of 28 g. The suspension was loaded into the RVA instrument and tested. The testing protocol included equilibration at 50 °C for 1 min, heating to 95 °C in 3.42 min, holding at 95 °C for 2.30 min, cooling to 50 °C in 3.48 min, and final equilibration at 50 °C for 2 min. The paddle initially rotated at 960 rpm for 10 s to ensure complete mixing and then maintained a constant speed of 160 rpm for the duration of the test.

### 2.8. Determination of Steady Shear Rheological Properties 

The fresh samples prepared by RVA were analyzed using a rheometer (AR1000-N, TA Instruments, Wilmington, DE, USA) equipped with a parallel plate geometry of 40 mm diameter and a gap size of 1000 μm, as described in a previous study [13]. Following this, the edges of pastes were covered with a low-density silicone oil and equilibrated for 5 min until reaching 25 °C. Power law ($\sigma =K\dot{\gamma }$^n^) was used to model the data, where σ is shear stress (Pa), K is consistency coefficient (Pa∙s^n^), $\dot{\gamma }$ is shear rate (s^−1^), and n is dimensionless flow behavior index.

### 2.9. Determination of Dynamic Rheological Properties

For dynamic rheological measurement, starch suspensions (20%, *w*/*w*) were prepared by adding the powder to distilled water, and mixed with a magnetic stirrer at 25 °C for 30 min and loaded onto a platform, which was then fitted between a 40 mm diameter geometry to a testing gap of 1000 mm. Starch suspensions were immediately conveyed to the rheometer and subjected to a temperature ramp from 25 to 95 °C, followed by cooling back to 25 °C at a scanning rate of 2 °C/min.

### 2.10. Determination of Gel Texture 

To obtain gels, the starch pastes that underwent RVA treatment were stored at a temperature of 4 °C for 24 h. Texture parameters were measured using a TA-XT2i Texture Analyser (Stable Micro Systems, Godalming, UK). The measurement followed a two-cycle program as described by Wang et al. [11]. The specific settings used were a distance of 10 mm, a speed of 1 mm/s and a cylindrical probe with a diameter of 15 mm.

### 2.11. Statistical Analysis

IBM-SPSS-20 (IBM, Armonk, NY, USA) software was used to carry out statistical data analysis. ANOVA was performed, and Duncan’s test was applied to determine statistical significance at a 95% confidence level (*p* ≤ 0.05).

## 3. Results and Discussion

### 3.1. Granule Size Distribution of Native and Acetylated Starches

**Figure 1:**
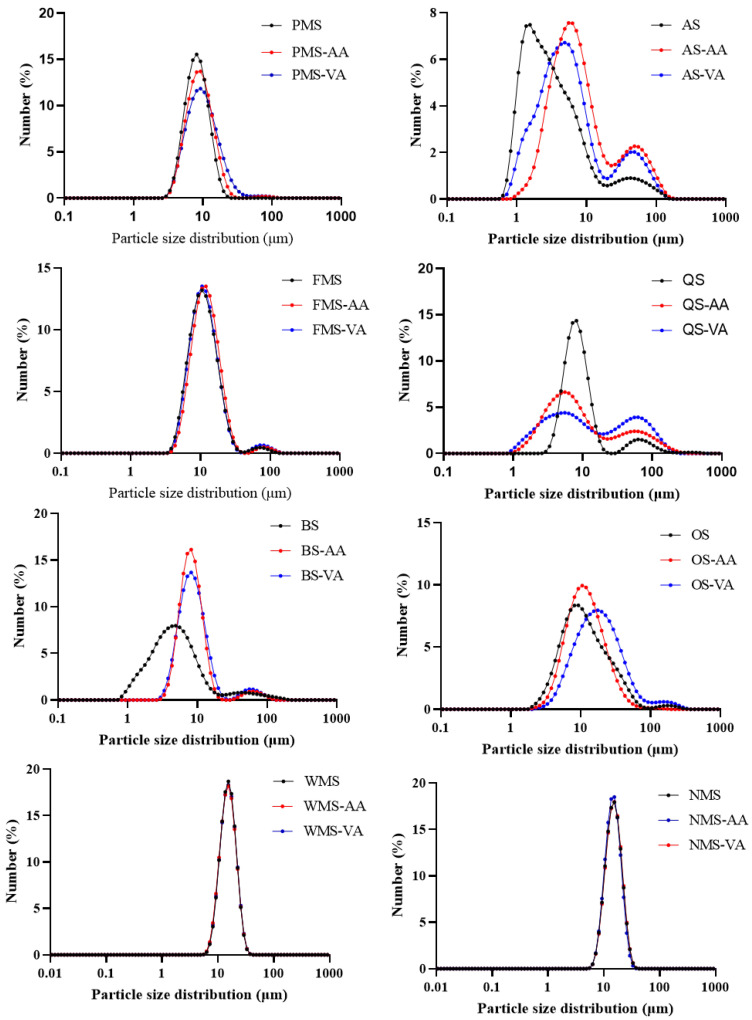
Starch granule size distribution of native, acetic anhydride and vinyl acetate modified starches.

The size distribution curves for coarse cereals starches before and after acetylation are displayed in Figure 1, while Table S1 summarizes the 10th (d10), medium (d50), 90th (d90) percentile and span factors of the granules. PMS exhibited a typical monomodal distribution of granule sizes, characterized by relatively smaller span factors, similar to WMS and NMS. On the other hand, AS, FMS, QS, BS, and OS displayed a visibly bimodal or broader distribution of granule sizes, with relatively larger span factors. This can be attributed to the aggregation of starch granules, as extensively documented in previous studies [14]. Noda et al. [15], proposed a more precise classification system for small granule starch, dividing it into two categories: small (5–10 μm) and very small (<5 μm). The average particle size (d50) of AS and BS was determined to be 2.66 and 4.48 μm, respectively, indicating that these starches consisted of very small granules. On the other hand, PMS and QS had d50 values of 8.15 and 8.32 μm, respectively, which aligned with the definition of small granules. Despite falling into the small granule category, FMS and OS still had smaller particle sizes (d50) of 10.8 and 10.6 μm compared to WMS and NMS.

For larger starch (WMS and NMS), both acetic anhydride and vinyl acetate modification did not significantly change their granule size. For six underutilized starches, both acetic anhydride and vinyl acetate modification induced a curve shift to the right or broadened granule size distribution to different extents. Moreover, the bimodal distribution of AS, QS, BS, and OS was more obvious after acetylation. Accordingly, the values of d10, d50, d90 increased after acetylation. The increased granule size of PMS, OS, QS and BS after vinyl acetate modification was greater than after acetic anhydride modification. This could be due to the acetyl groups when esterified to starch chains, with the hydrophobic end spreading out from the granule surface and providing a comparable hydrophobic interaction [16], resulting in greater extent of granule aggregation, thereby increasing granule size [17].

Granule size of AS was smaller than that of PMS, QS, FMS, and OS, but AS had lower DS using vinyl acetate than QS, FMS, and OS. Although six underutilized starches showed smaller granule size than WMS and NMS, DS value of acetic anhydride modified OS was lower than that of WMS and NMS; meanwhile, DS values of vinyl acetate modified PMS and AS were also lower than those of WMS and NMS. Moreover, starch granule size was not significantly related to DS values as indicated by correlation analysis (Table S2). These results indicated that granule size did not significantly affect acetylation efficiency in starches from different plant origins.

### 3.2. Pasting Properties of Native and Acetylated Starches

**Figure 2:**
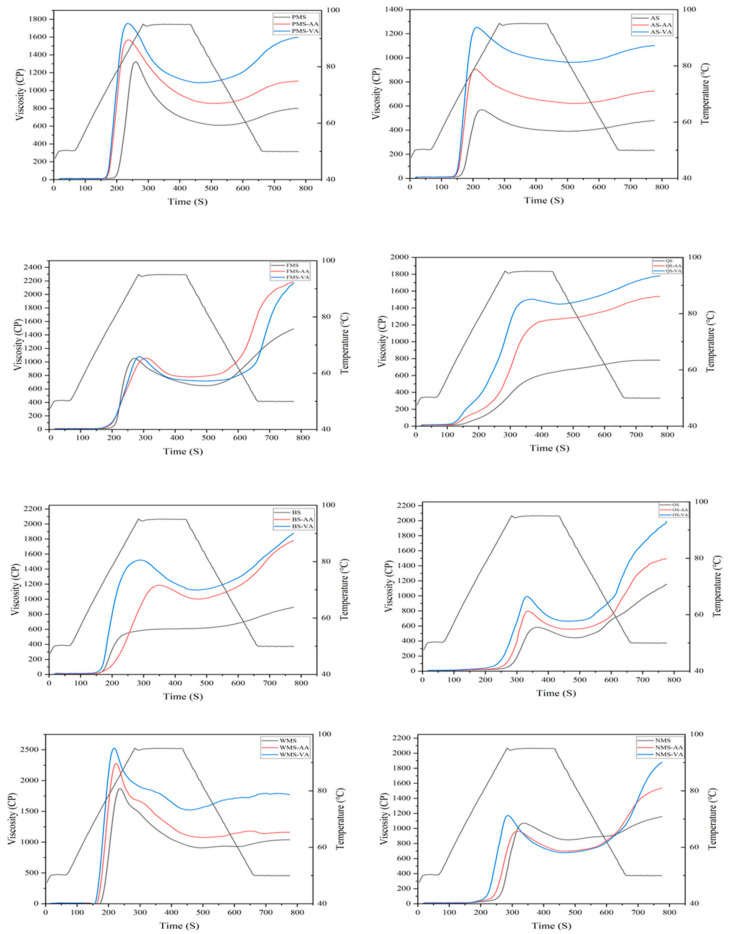
RVA profiles of native, acetic anhydride, and vinyl acetate modified proso millet, amaranth, foxtail millet, quinoa, buckwheat, oat, waxy maize, and normal maize starches.

The pasting curves are presented in Figure 2 and viscosity parameters are summarized in Table 2. During the heating phase, starch granules expand and rupture resulting in the breakdown of hydrogen bonds. The paste consists of a molecular dispersion of dissolved starch molecules and discontinuous phase of swollen granules and granule fragments [18]. Increased viscosity is thus observed during the heating process. When a large number of granules swell, the peak viscosity is reached during gelatinization. As the process progresses, the crystalline regions of amylopectin chains in starch granules disrupt, and the amylopectin chains are released from the swollen granules into the solution, leading to a decrease in viscosity. During the cooling stage, the viscosity increases, which indicates the tendency of the amylose present in the hot paste to re-associate with decrease in temperature [19].

Peak viscosity relates to the extent to which starch granules can swell and shear, indicating their water-holding capacity [19]. For native underutilized starch, the order of peak viscosity was PMS, FMS, OS, QS, BS, and AS. Higher setback viscosities indicated a higher tendency of the starch granules to retrograde [20]. For native starch, the order of setback was FMS, OS, BS QS, PMS, and AS. Non-waxy starch exhibited higher setback than waxy. The primary reason for experiencing high setback is attributed to a higher level of amylose content. This is primarily due to the linear chain structure of amylose molecules, which enables them to readily reform hydrogen bonds in comparison to amylopectin molecules [21].

As illustrated in Figure 2, the pasting profile of acetylated starch was higher than native starch, except for FMS and NMS. For PMS, AS, BS, and OS, acetylation generally increases the peak, final, and setback viscosities. Moreover, the incorporation of acetyl groups disrupts the organized structures of starch, resulting in an increase in the proportion of amorphous starch structures. Consequently, the disintegrated starch granules are more susceptible to breakdown [22], resulting in a lower pasting temperature (Table 2). Due to the higher water-holding capacity of starch amorphous structures compared to ordered structures, the water-holding capacity increased after acetylation [23]. This increase was accompanied by a higher peak viscosity. Similar findings were reported for rice and potato starches, which showed a higher peak viscosity after acetylation with acetic anhydride and vinyl acetate [24,25]. Starches that exhibit high peak viscosity are highly desirable as thickeners, and incorporating a small amount of acetylated starch can effectively substitute for larger quantities of native starch [26]. Additionally, acetylation did not greatly increase the peak viscosity of FMS and NMS, which may be due to the maintenance of ordered structure in FMS and NMS. The higher amylose content of starch led to tighter structure with compact molecular packing [27], with acetyl groups rarely disorganizing their structure.

Starches modified with vinyl acetate exhibited a higher peak viscosity compared to those acetylated with acetic anhydride using the same starch. Vinyl acetate reagent could penetrate internal structures while acetic anhydride reacted on starch granule surface [7]. Compared to acetic anhydride, vinyl acetate modification may cause more ordered structures to convert to less ordered structures. This modification enhances the starch molecules’ capacity to establish hydrogen bonds with water, leading to an increase in peak viscosity. Vinyl acetate modified starch with high viscosity would serve as a new type of alternative thickening agent and stabilizing agent in yogurt or salad dressings to improve the quality properties of final products.

Moreover, six underutilized starches after acetic anhydride and vinyl acetate modification showed an increase in final and setback viscosities, which was consistent with previous studies [27]. However, some studies reported a reduction in setback after acetylation [28]. The decrease in setback viscosity may be attributed to steric hindrance caused by the introduction of acetyl groups, as higher degrees of substitution (DS) could hinder the close parallel alignment of starch molecules, thereby reducing setback viscosity [29]. In this study, DS of acetylated starches was relatively low, which enhanced viscosity properties by improving the capacity of starch molecules to form hydrogen bonds with water [22].

Both acetic anhydride and vinyl acetate modification increased peak and final viscosity of starches. Regardless of whether the DS value of vinyl acetate starch was high or low, vinyl acetate exhibited a more pronounced effect than acetic anhydride. For acetic anhydride or vinyl acetate modified starches, correlation analysis result showed that peak viscosity, breakdown, final viscosity, and setback were not related with degree of substitution (Tables S3 and S4). Therefore, the acetylation reagent type was the major factor determining the pasting properties of acetylated starch.

### 3.3. Shear Flow Properties of Native and Acetylated Starches

**Figure 3:**
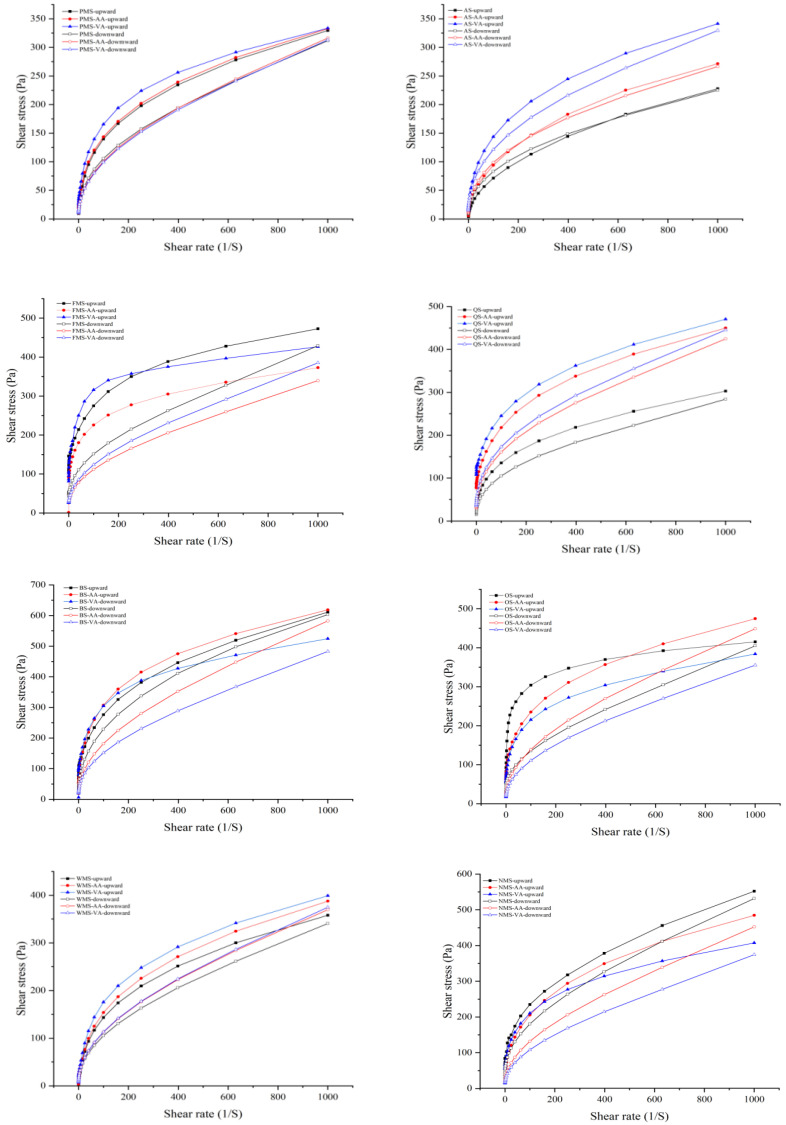
Steady shear flow behavior of native, acetic anhydride and vinyl acetate modified starches during steady shear from 0.1 to 1000 s^−1^ and 1000 to 0.1 s^−1^.

As shown in Figure 3, the starch pastes before and after modification by acetic anhydride or vinyl acetate exhibit a typical non-Newtonian (pseudoplastic) behavior, indicating a high shear-thinning behavior where the starch network tends to breakdown under higher shear rates [30]. The shear-thinning behavior might be attributed to increased breaking of the intra- and intermolecular associative bonding system in the starch network micelles because of shearing at higher shear rates [19].

The values for the consistency coefficient (K) and flow index (n) can be found in Table 3. All starches had n values below 1, indicating pseudoplasticity. A higher K value signifies a greater viscosity. The low n values suggest that the starches had a more pronounced pseudoplastic, shear-thinning behavior [13]. Both acetic anhydride or vinyl acetate modification increased K values and decreased n values of PMS, AS, and QS, indicating that acetylation increased viscosity and pseudoplasticity. The extent of vinyl acetate modification was greater than acetic anhydride. The higher K values of acetylated starches was consistent with other reports [31,32]. Interestingly, for non-waxy starches, acetic anhydride modification decreased K and increased n values of FMS, BS, OS, and NMS, showing a less thickening, more thinning effect. Acetic anhydride modification enhanced the flow properties of FMS, BS, OS, and NMS, and the intermolecular disruption rate was higher during shearing. Acetic anhydride is more likely to attack amylose than amylopectin [8]. The increased hindrance caused by acetyl groups enhances the flow properties of amylose, leading to a decrease in the strength of the forces between the starch granules and the shear thinning behavior [16].

The K values for the upward curves of PMS, FMS, QS, BS, and OS were higher than in the downward curves, indicating thixotropy in these starches. This is because as the fluid is subjected to gradually increasing shear stress, the structure of the starch paste is gradually weakened [33]. When the shear stress is reduced, the structure begins to recover, but the speed of recovery is different from the speed of disruption, resulting in the formation of a hysteresis loop [34]. The area of the hysteresis loop represents the strength of thixotropy, which reflects the ability of the gel structure to recover under shear force [35]. According to Figure 3, acetylated starch had the larger hysteresis loop, exhibiting a stronger thixotropy and poorer recovery ability compared to native starch. Firstly, the introduction of acetyl groups inhibited hydrogen bond formation both between starch molecules and between starch and water, weakening intermolecular forces and resulting in stronger thixotropy and poorer recovery ability. Secondly, acetyl groups introduced steric hindrance, preventing starch molecular chains from re-forming tightly packed structures, which decreased recovery ability [25]. As a result, the rearrangement and reassociation of starch molecules become more difficult. It is worth noting that AS exhibited an intriguing phenomenon where the downward curves had larger K values than the upward curves in the initial anticlockwise loops. This phenomenon is known as the anti-thixotropy of starch during shearing, as described by Dewar and Joyce [36]. The findings suggested that the reformation of intermolecular interactions in AS occurred at a faster rate than their disruption during shearing. However, this anti-thixotropy behavior disappeared after acetylation. The introduction of acetyl groups into the starch molecules disrupted these interactions through steric hindrance and hydrophobic effects [30]. Consequently, the reformation of intermolecular interactions was significantly slowed or prevented, leading to the elimination of anti-thixotropy.

### 3.4. Dynamic Oscillatory Properties of Native and Acetylated Starches

The dynamic rheological tests provide valuable information on the viscoelastic properties of starch pastes without disrupting their structures [31]. Storage modulus (G′), loss modulus (G″) and the loss factor (tan δ), defined by the G″/G′ ratio of starch pastes in heating and cooling process are summarized in Table 4. G′, which is a key factor indicating the elasticity of starch pastes, is primarily responsible for reflecting their dynamic rheological properties [13]. As the starch pastes are heated, G′ shows a notable increase until it reaches its highest value (T_G′max_), after which it gradually decreases. This decline in G′ could be attributed to the melting of the crystallite, resulting in a softening of the starch granules [37]. During cooling, G′ increased as temperature decreased due to rearrangement of starch chains and formation of starch gel. Zheng et al. [38] believed that the amylose leaching out from swollen starch and then intertwining played a dominant role in increase in G′. Both acetic anhydride and vinyl acetate modification prominently decreased T_G′max_ on all starches, which was in line with the pasting temperature (Table 2). However, tan δ_G′max_ of coarse cereals starch decreased after acetylation, indicating that acetylated starch pastes are more elastic than viscous.

For waxy starch (PMS and AS), the acetylated gels exhibited higher G′_25°C_ and lower tan δ25 °C than their native counterparts, indicating that acetylation resulted in a firmer gel by enhancing retrogradation. While, non-waxy starch (FMS, QS, BS, and OS) showed opposite trends. These results suggested that the acetyl groups mainly retarded the establishment of hydrogen bonds between amylose molecules, and their steric hindrance decreased the reassociation of amylose. This effect decreases gel rigidity to increase the tan δ_25°C_ value. When starch lacked amylose, the rearrangement of amylopectin was slow because its highly branched molecule restricted water mobility and the opportunity to form hydrogen bonds [13]. The introduction of acetyl groups resulted in the formation of a stronger gel in waxy starch, which may be due to the increase in the number of entanglement points and the formation of junction zones between amylopectin chains. No matter whether acetylation decreased tan δ_25°C_ in waxy starch or increased tan δ_25°C_ in non-waxy starch, the extent of change in vinyl acetate modification was more pronounced than that of acetic anhydride.

**Figure 4:**
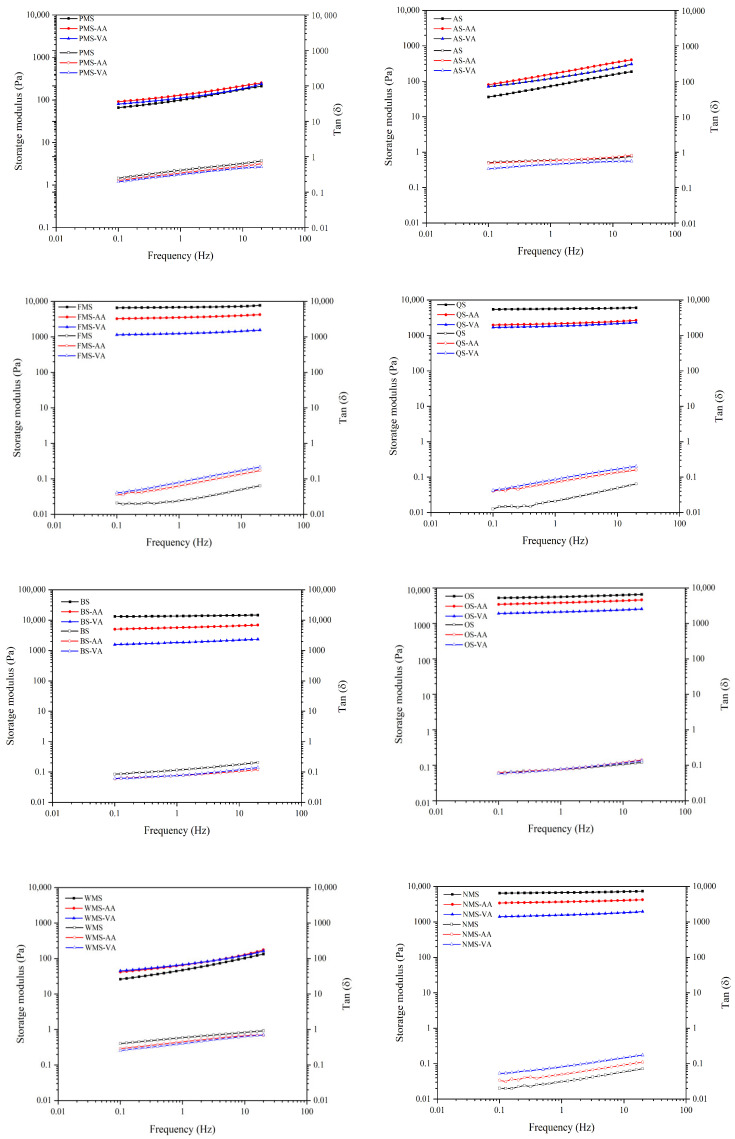
Storage modulus (G′) and tan δ (the ratio of G′ and G′′) of native, acetic anhydride and vinyl acetate modified starches in dynamic frequency sweep (from 0.1 and 20 Hz).

In the frequency range of 0.1 to 20 Hz, the values of G′ and tan δ (Figure 4) indicate the viscoelastic properties of gels. G′ represents the elasticity, specifically the short-term retrogradation of starch [13]. A higher G′ and lower tan δ indicate a solid and elastic gel network structure [19]. After acetylation, the G′ decreased and tan δ increased for FMS, QS, BS, OS, and NMS in the frequency range of 0.1 to 20 Hz. This suggests that acetylation resulted in a less solid and elastic gel network. Additionally, vinyl acetate modification had a greater impact on the values of G′ and tan δ in the frequency range of 0.1 to 20 Hz compared to acetic anhydride modification, primarily due to its higher DS. The DS values of vinyl acetate modified FMS, QS, BS, OS, and NMS were 0.073, 0.077, 0.074, 0.063, and 0.061, respectively. These values were generally higher than those of acetic anhydride modified FMS, QS, BS, OS, and NMS, which were 0.059, 0.063, 0.062, 0.044, and 0.058, respectively. The increased number of acetyl groups introduced into the starch molecules disrupted hydrogen bonding more effectively, resulting in more pronounced changes in viscoelastic properties of gels.

### 3.5. Texture Properties of Native and Acetylated Starch Gels

The textural properties of starch gels are important to predict the product behavior during formulation and storage, as well as optimizing industrial processes. The starch gel is formed by the reassociation of linear amylose and branched amylopectin chains after starch gelatinization. The hardness can characterize the strength of the gel network structure, that is, the strength of the gel to resist external forces [39]. For waxy starch (PMS and AS), vinyl acetate modification increased hardness (Table 5). For non-waxy starch (FMS, QS, BS, and OS), both acetic anhydride and vinyl acetate modification decreased hardness. The higher the amylose content, the stronger the gel hardness, mainly due to the retrogradation of starch gel caused by the crystallization of amylose [25]. The addition of acetyl groups increases the gap among amylose chains and decreases the formation of hydrogen bonds between their molecules. Therefore, the gel strength of starch network structure was weakened after acetylation. However, gel hardness of vinyl acetate modified waxy underutilized starches increased, indicating the increased re-association of amylopectin molecules. There were more acetyl groups in amylopectin of vinyl acetate acetylated starch [8], and hydrophobic acetylated amylopectin chains are more prone to entanglement and formation of hydrogen bonds.

Cohesiveness measures the degree to which the gel structure is disrupted during initial compression [40]. It provides an indication of the sample’s ability to withstand deformation. The addition of both acetic anhydride and vinyl acetate to modified PMS, FMS, QS, and BS increased the cohesiveness of the samples (Table 5). The primary factor in the formation of a cohesive starch gel is the leaching out of amylose chains from acetylated granules and their subsequent reassociation [39]. Gumminess, on the other hand, refers to the energy required to break a semi-solid sample into a stable state when consumed. It reflects not only the strength of the gel network but also the extent of internal bonding within the gel. Acetylation led to a decrease in gumminess values for gels, with the exception of PMS. The decrease in gumminess for most acetylated gels was due to the disruption of hydrogen bonding and gel network integrity caused by acetyl groups [41]. Waxy PMS are composed almost entirely of amylopectin. In the case of acetylated PMS, the acetyl groups were primarily incorporated into the amylopectin chains, increasing their hydrophobicity and promoting entanglement and hydrogen bonding between amylopectin molecules [42]. This enhanced re-association of amylopectin led to the formation of a stronger gel network [18], which increased the gumminess value in acetylated PMS. Additionally, the texture properties of WMS, WMS-AA, and WMS-VA could not be measured because their pastes, stored at 4 °C for 24 h, either failed to form gels or the gels formed were too soft.

## 4. Conclusions

In this study, acetic anhydride and vinyl acetate differentially altered the pasting and rheological properties of two waxy and four non-waxy underutilized starches under low DS (DS < 0.1). The results of DS and granule size of six starches indicated that granule size did not significantly affect acetylation efficiency among starches of different plant origin. Both acetic anhydride and vinyl acetate modifications increased peak and final viscosity. Regardless of whether the DS value of vinyl acetate starch was high or low, vinyl acetate exhibited a more pronounced effect than acetic anhydride. Acetic anhydride modification decreased K and increased n values of non-waxy starch, but these results were the opposite for waxy starch. Acetylated non-waxy starch showed lower gel hardness and higher tan δ in frequency sweep, indicating a less solid and less elastic gel network. Vinyl acetate modification increased gel hardness and decreased tan δ for waxy starch, resulting in a firmer gel by enhancing the retrogradation. Therefore, the presence or absence of amylose would influence the rheological and gel properties of acetic anhydride and vinyl acetate modified starches, regardless of the source of the starch. The possible mechanisms underlying the different influence of acetic anhydride and vinyl acetate on starch gels may be proposed: (1) The steric hindrance of acetyl groups prevents close association of amylose chains and thus hinders the hydrogen bond formation of non-waxy starches, thereby strengthening shear thinning behavior and reducing the rigidity of the gel; (2) the acetyl groups of vinyl acetate acetylated starch are more prevalent in amylopectin; hydrophobic acetylated amylopectin chains are more prone to entanglement and formation of junction zones than native amylopectin with highly branched molecules, thereby leading to a stronger gel in waxy starch. The findings highlight the potential of acetylation to tailor starch properties for specific applications, such as improving viscosity or texture in food and industrial products. Future research should explore practical applications of acetylated underutilized starches in food systems, as well as their interactions with other components, to further advance the understanding and utilization of these materials.

## Figures and Tables

**Table 1:** Information for native and acetylated starches [9].

Samples	Abbreviation	Amylose Content (%)	Acetic Anhydride Modified Starches	Degree of Substitution (DS)	Vinyl Acetate Modified Starches	Degree of Substitution (DS)
Proso millet starch	PMS	3.82 ± 0.63 d	PMS-AA	0.061 ± 0.002 abc	PMS-VA	0.039 ± 0.002 d
Amaranth starch	AS	1.11 ± 0.07 f	AS-AA	0.069 ± 0.004 a	AS-VA	0.052 ± 0.004 c
Foxtail millet starch	FMS	29.3 ± 0.26 b	FMS-AA	0.059 ± 0.002 bc	FMS-VA	0.073 ± 0.002 a
Quinoa starch	QS	23.82 ± 0.09 c	QS-AA	0.063 ± 0.002 abc	QS-VA	0.077 ± 0.001 a
Buckwheat starch	BS	32.2 ± 0.30 a	BS-AA	0.062 ± 0.001 abc	BS-VA	0.074 ± 0.001 a
Oat starch	OS	23.0 ± 0.60 c	OS-AA	0.044 ± 0.003 d	OS-VA	0.063 ± 0.002 b
Waxy maize starch	WMS	2.45 ± 0.04 e	WMS-AA	0.067 ± 0.002 ab	WMS-VA	0.058 ± 0.001 bc
Normal maize starch	NMS	32.4 ± 0.30 a	NMS-AA	0.058 ± 0.001 c	NMS-VA	0.061 ± 0.004 b

Mean ± SD values from triplicate data followed by different lowercase letters in the same column are significantly different (*p* < 0.05).

**Table 2:** Pasting properties of native, acetic anhydride, and vinyl acetate modified starches.

Sample	Peak Viscosity (cP)	Breakdown (cP)	Final Viscosity (cP)	Setback (cP)	Pasting Temperature (℃)
PMS	1318 ± 8.5 Hc	713 ± 2.1 Da	800 ± 2.1 Lc	191 ± 4.24 Mc	77.7 ± 1.2 Ga
PMS-AA	1563 ± 5.7 Eb	710 ± 3.5 Da	1108 ± 2.8 Ib	255 ± 4.95 Lb	72.8 ± 1.2 IJb
PMS-VA	1753 ± 2.1 Da	663 ± 1.4 Eb	1601 ± 4.9 Da	511 ± 1.41 Ia	71.9 ± 0.1 JKLb
AS	568 ± 2.1 Tc	180 ± 1.4 Nb	476 ± 4.2 Nc	89 ± 0.71 Oc	71.9 ± 0.1 JKLa
AS-AA	901 ± 7.8 Pb	280 ± 7.8 JKa	724 ± 0.7 Mb	103 ± 0.71 Ob	68.8 ± 0.1 Mb
AS-VA	1252 ± 1.4 Ia	290 ± 2.1 Ja	1101 ± 2.1 Ia	138 ± 2.83 Na	68.6 ± 0.0 Mb
FMS	1052 ± 2.1 Mb	399 ± 7.1 Ga	1500 ± 15.6 Fb	848 ± 10.61 Fc	80.8 ± 0.0 Fa
FMS-AA	1056 ± 2.1 LMb	274 ± 7.8 JKc	2176 ± 13.4 Aa	1403 ± 5.66 Cb	78.3 ± 0.0 Gb
FMS-VA	1080 ± 2.1 Ka	364 ± 5.7 Hb	2178 ± 12.0 Aa	1462 ± 15.56 Ba	78.3 ± 0.0 Gb
QS	629 ± 2.1 Rc	57 ± 2.8 Rb	784 ± 3.5 Lc	212 ± 1.41 Mc	94.5 ± 0.0 Aa
QS-AA	1254 ± 4.9 Ib	80 ± 4.2 Pa	1532 ± 8.5 Eb	359 ± 0.71 Ja	88.8 ± 0.0 Db
QS-VA	1501 ± 4.2 Ga	64 ± 6.4 PQb	1776 ± 6.4 Ca	338 ± 4.24 Jb	66.1 ± 0.1 Nc
BS	605 ± 3.5 Sc	3 ± 0.0 Sc	887 ± 7.8 Kc	285 ± 4.24 Kc	73.5 ± 0.0 Ib
BS-AA	1182 ± 5.7 Jb	184 ± 3.5 Nb	1775 ± 8.5 Cb	777 ± 6.36 Ga	78.3 ± 0.0 Ga
BS-VA	1529 ± 10.6 Fa	402 ± 4.9 Ga	1883 ± 8.5 Ba	756 ± 2.83 GHb	71.2 ± 0.1 Lc
OS	635 ± 3.5 Rc	151 ± 5.6 Oc	1229 ± 11.3 Gc	746 ± 13.44 Hc	94.9 ± 0.1 Aa
OS-AA	800 ± 4.9 Qb	242 ± 4.2 Lb	1494 ± 1.4 Fb	937 ± 2.12 Eb	92.9 ± 0.0 Bb
OS-VA	994 ± 0.0 Na	323 ± 11.3 Ia	2174 ± 14.9 Aa	1503 ± 25.5 Aa	88.1 ± 0.1 Dc
WMS	1939 ± 28.3 Cc	1037 ± 35.4 Cb	1041 ± 4.2 Jc	139 ± 11.31 Nb	74.4 ± 0.0 Ha
WMS-AA	2320 ± 12.7 Bb	1245 ± 4.9 Aa	1168 ± 11.3 Hb	93 ± 3.54 Oc	72.1 ± 0.1 JKb
WMS-VA	2545 ± 6.4 Aa	1061 ± 12.7 Bb	1771 ± 0.7 Ca	287 ± 7.07 Ka	71.5 ± 0.6 Lb
NMS	1070 ± 2.1 KLb	218 ± 6.4 Mc	1150 ± 10.6 Hc	298 ± 14.85 Kc	90.9 ± 0.7 Ca
NMS-AA	956 ± 9.2 Oc	260 ± 4.2 KLb	1543 ± 6.4 Eb	847 ± 11.31 Fb	88.0 ± 0.0 Db
NMS-VA	1174 ± 2.8 Ja	492 ± 2.8 Fa	1868 ± 20.5 Ba	1186 ± 26.16 Da	83.2 ± 0.0 Ec

Mean ± SD values from triplicate data followed by different lowercase letters of the same origin and different uppercase letters in the same column are significantly different (*p* < 0.05).

**Table 3:** Power Law parameters of native, acetic anhydride and vinyl acetate modified starches.

Samples	Upward Curve			Downward Curve		
	K (Pa⋅s^n^)	n	R^2^	K (Pa⋅s^n^)	n	R^2^
PMS	19.5 ± 0.1 Jc	0.41 ± 0.00 Ca	0.99	13.0 ± 0.0 KLa	0.46 ± 0.00 Eb	0.99
PMS-AA	24.6 ± 0.1 Ib	0.38 ± 0.01 Db	0.99	12.0 ± 0.5 LMab	0.47 ± 0.00 Da	0.99
PMS-VA	33.5 ± 0.8 Ha	0.34 ± 0.00 Fc	0.99	11.2 ± 0.1 Mb	0.48 ± 0.00 Ca	0.99
AS	7.4 ± 0.0 Kc	0.47 ± 0.03 Aa	0.99	12.5 ± 0.1 KLMc	0.42 ± 0.00 Ga	0.99
AS-AA	11.8 ± 0.3 Kb	0.46 ± 0.01 Aab	0.99	15.8 ± 1.3 Jb	0.41 ± 0.00 Hb	0.99
AS-VA	19.1 ± 0.0 Ja	0.41 ± 0.00 Cb	0.99	23.4 ± 0.2 Fa	0.39 ± 0.00 IJc	0.99
FMS	115.9 ± 2.3 Aa	0.19 ± 0.01 Mab	0.97	30.2 ± 1.2 Da	0.37 ± 0.00 LMc	0.97
FMS-AA	83.7 ± 0.1 Db	0.22 ± 0.00 La	0.97	17.4 ± 0.1 Ib	0.42 ± 0.00 Gb	0.98
FMS-VA	118.2 ± 2.3 Aa	0.18 ± 0.02 Mb	0.99	17.2 ± 0.7 Ib	0.44 ± 0.00 GFa	0.98
QS	35.5 ± 0.1 Hc	0.30 ± 0.00 GHa	0.98	17.6 ± 0.1 Ic	0.39 ± 0.00 IJa	0.99
QS-AA	70.2 ± 2.5 Eb	0.26 ± 0.00 JKb	0.96	30.4 ± 0.2 Db	0.37 ± 0.00 Lb	0.99
QS-VA	100.8 ± 4.8 Ba	0.21 ± 0.01 Lc	0.94	35.2 ± 0.1 Ba	0.36 ± 0.00 Mc	0.99
BS	79.9 ± 5.0 Db	0.29 ± 0.01 HIb	0.98	38.3 ± 2.0 Aa	0.40 ± 0.01 IJc	0.99
BS-AA	67.4 ± 0.6 Eb	0.32 ± 0.01 FGa	0.99	20.4 ± 0.3 Gb	0.48 ± 0.00 Ca	0.99
BS-VA	94.8 ± 5.8 Ca	0.25 ± 0.01 Kc	0.99	19.0 ± 0.6 Hb	0.46 ± 0.00 DEb	0.99
OS	118.0 ± 3.0 Aa	0.18 ± 0.02 Mb	0.97	25.4 ± 1.0 Ea	0.39 ± 0.00 Jb	0.99
OS-AA	68.3 ± 0.7 Eb	0.28 ± 0.00 IJa	0.99	16.5 ± 0.1 IJb	0.47 ± 0.00 Da	0.97
OS-VA	71.8 ± 3.3 Eb	0.25 ± 0.01 Ka	0.99	13.6 ± 0.1 Kc	0.47 ± 0.00 Da	0.99
WMS	17.8 ± 0.4 Jb	0.44 ± 0.00 Ba	0.99	11.3 ± 0.1 Ma	0.49 ± 0.00 Bb	0.99
WMS-AA	19.5 ± 1.1 Jb	0.44 ± 0.00 Ba	0.99	11.9 ± 0.3 LMa	0.50 ± 0.00 Aa	0.99
WMS-VA	26.8 ± 0.6 Ia	0.40 ± 0.00 CDb	0.98	11.5 ± 0.0 Ma	0.50 ± 0.00 Aa	0.99
NMS	60.1 ± 1.0 Fa	0.31 ± 0.00 Gb	0.99	31.8 ± 0.1 Ca	0.40 ± 0.00 Ib	0.98
NMS-AA	40.5 ± 1.6 Gb	0.36 ± 0.01 Ea	0.99	13.5 ± 0.1 Kb	0.50 ± 0.00 Aa	0.99
NMS-VA	59.7 ± 0.6 Fa	0.28 ± 0.00 IJc	0.99	11.2 ± 0.1 Mc	0.50 ± 0.00 Aa	0.99

Mean ± SD values from triplicate data followed by different lowercase letters of the same origin and different uppercase letters in the same column are significantly different (*p* < 0.05).

**Table 4:** Dynamic rheological properties of native, acetic anhydride and vinyl acetate modified starches.

Samples	Heating Process					Cooling Process	
	T_G′max_ (°C)	G′_max_ (kPa)	tan δ_G′max_	G′_95°C_ (kPa)	tan δ_95°C_	G′_25°C_ (kPa)	tan δ_25°C_
PMS	75.1 ± 0.2 ^Ea^	659 ± 5 ^JKc^	0.257 ± 0.006 ^Fa^	66 ± 1 ^Hc^	0.340 ± 0.004 ^Da^	98 ± 1 ^Kb^	0.433 ± 0.017 ^Ba^
PMS-AA	69.2 ± 0.4 ^HIb^	857 ± 65 ^Ja^	0.197 ± 0.005 ^Hb^	135 ± 8 ^Ha^	0.204 ± 0.010 ^Ic^	135 ± 6 ^Ka^	0.342 ± 0.004 ^Db^
PMS-VA	69.4 ± 0.5 ^HIb^	766 ± 29 ^Jab^	0.173 ± 0.005 ^Ic^	104 ± 4 ^Hb^	0.218 ± 0.003 ^Hb^	120 ± 11 ^Kab^	0.322 ± 0.008 ^Eb^
AS	83.7 ± 0.45 ^Bc^	48 ± 2 ^Lc^	0.526 ± 0.001 ^Aa^	37 ± 1 ^Hc^	0.588 ± 0.011 ^Aa^	36 ± 1 ^Kc^	0.589 ± 0.013 ^Aa^
AS-AA	69.4 ± 1.3 ^HIb^	190 ± 11 ^KLa^	0.435 ± 0.026 ^Cb^	89 ± 3 ^Ha^	0.534 ± 0.005 ^Bb^	162 ± 11 ^Ka^	0.596 ± 0.026 ^Aa^
AS-VA	87.9 ± 0.2 ^Aa^	77.82 ± 2 ^Lb^	0.315 ± 0.001 ^Dc^	74 ± 4 ^Hb^	0.321 ± 0.005 ^Ec^	101 ± 6 ^Kb^	0.437 ± 0.023 ^Bb^
FMS	76.5 ± 0.0 ^Da^	6560 ± 380 ^Fb^	0.111 ± 0.001 ^La^	1376 ± 59 ^Da^	0.109 ± 0.000 ^Oa^	6552 ± 193 ^Ba^	0.026 ± 0.001 ^Jc^
FMS-AA	71.7 ± 0.3 ^Gb^	10130 ± 196 ^Ba^	0.077 ± 0.001 ^Nb^	1275 ± 90 ^Da^	0.107 ± 0.016 ^Oa^	3342 ± 81 ^Eb^	0.069 ± 0.002 ^HIb^
FMS-VA	69.7 ± 0.3 ^HIc^	7287 ± 212 ^Eb^	0.071 ± 0.002 ^Nc^	637 ± 41 ^Gb^	0.116 ± 0.013 ^NOa^	1133 ± 100 ^Jc^	0.085 ± 0.001 ^GHa^
QS	68.9 ± 0.1 ^IJa^	5593 ± 302 ^Gc^	0.070 ± 0.001 ^Na^	2109 ± 15 ^Ba^	0.087 ± 0.002 ^Pab^	5346 ± 244 ^Ca^	0.024 ± 0.001 ^Jc^
QS-AA	64.2 ± 0.2 ^Lb^	6431 ± 213 ^Fb^	0.055 ± 0.001 ^Ob^	1621 ± 11 ^Cb^	0.084 ± 0.000 ^Pb^	1991 ± 13 ^FGb^	0.082 ± 0.002 ^GHb^
QS-VA	63.0 ± 0.3 ^Mc^	8108 ± 47 ^CDa^	0.056 ± 0.000 ^Ob^	1521 ± 9 ^Cc^	0.093 ± 0.003 ^PQa^	1713 ± 84 ^GHb^	0.091 ± 0.002 ^GHa^
BS	71.1 ± 0.0 ^Ga^	5916 ± 97 ^Gb^	0.152 ± 0.009 ^Ja^	2997 ± 165 ^Aa^	0.163 ± 0.002 ^Ja^	13072 ± 332 ^Aa^	0.031 ± 0.001 ^Jc^
BS-AA	66.4 ± 0.5 ^Kb^	11458 ± 727 ^Aa^	0.100 ± 0.000 ^LMc^	3078 ± 124 ^Aa^	0.106 ± 0.000 ^OPc^	5042 ± 136 ^Db^	0.096 ± 0.001 ^Gb^
BS-VA	64.8 ± 0.2 ^Lc^	5692 ± 272 ^Gbc^	0.130 ± 0.006 ^Kb^	1069 ± 51 ^Eb^	0.130 ± 0.005 ^Mb^	1677 ± 92 ^HIc^	0.124 ± 0.001 ^Fa^
OS	71.3 ± 0.2 ^Ga^	7622 ± 29 ^DE^	0.094 ± 0.000 ^Ma^	1080 ± 9 ^Eb^	0.110 ± 0.003 ^Ob^	2006 ± 48 ^Fb^	0.081 ± 0.002 ^GHc^
OS-AA	65.7 ± 0.1 ^Kb^	10963 ± 44 ^Aa^	0.051 ± 0.000 ^Oc^	2097 ± 76 ^Ba^	0.118 ± 0.008 ^NOb^	1669 ± 32 ^HIc^	0.123 ± 0.003 ^Fa^
OS-VA	70.1 ± 1.1 ^Ha^	8541 ± 308 ^Cb^	0.076 ± 0.002 ^Nb^	2010 ± 75 ^Ba^	0.153 ± 0.002 ^KLa^	5352 ± 83 ^Ca^	0.097 ± 0.001 ^Gb^
WMS	71.3 ± 0.1 ^Ga^	505 ± 13 ^JKL^	0.276 ± 0.000 ^Eb^	33 ± 2 ^Hb^	0.358 ± 0.002 ^Ca^	42 ± 6 ^Kb^	0.600 ± 0.021 ^Aa^
WMS-AA	68.2 ± 0.1 ^Jb^	866 ± 11 ^J^	0.238 ± 0.001 ^Gc^	65 ± 3 ^Ha^	0.272 ± 0.006 ^Fb^	65 ± 1 ^Ka^	0.451 ± 0.006 ^Bb^
WMS-VA	66.6 ± 0.3 ^Kc^	562 ± 23 ^JKL^	0.474 ± 0.002 ^Ba^	70 ± 1 ^Ha^	0.236 ± 0.004 ^Gc^	66 ± 1 ^Ka^	0.402 ± 0.001 ^Cc^
NMS	78.6 ± 0.2 ^Ca^	4888 ± 374 ^Hab^	0.096 ± 0.002 ^Mb^	2142 ± 87 ^Ba^	0.093 ± 0.003 ^PQc^	6698 ± 377 ^Ba^	0.032 ± 0.000 ^Jc^
NMS-AA	74.2 ± 0.5 ^Fb^	5777 ± 434 ^Ga^	0.106 ± 0.001 ^LMa^	1386 ± 79 ^Db^	0.127 ± 0.001 ^Mb^	3420 ± 164 ^Eb^	0.058 ± 0.002 ^Ib^
NMS-VA	73.3 ± 0.2 ^Fb^	4324 ± 51 ^Ib^	0.108 ± 0.000 ^LMa^	654 ± 9 ^Gc^	0.149 ± 0.001 ^La^	1402 ± 52 ^IJc^	0.092 ± 0.000 ^Ga^

Mean ± SD values from triplicate data followed by different lowercase letters of the same origin and different uppercase letters in the same column are significantly different (*p* < 0.05).

**Table 5:** Texture properties of native, acetic anhydride, and vinyl acetate-modified starches gels.

Samples	Hardness (g)	Adhesiveness (g.s)	Springiness	Cohesiveness	Gumminess
PMS	1.10 ± 0.05 ^Jb^	−2.48 ± 0.14 ^Gb^	2.66 ± 0.04 ^ABa^	0.93 ± 0.04 ^CDEb^	1.04 ± 0.02 ^Kb^
PMS-AA	1.11 ± 0.00 ^Jb^	−3.32 ± 0.45 ^Ha^	2.50 ± 0.04 ^Ba^	0.99 ± 0.08 ^BCDEb^	1.11 ± 0.09 ^Kb^
PMS-VA	1.25 ± 0.03 ^Ja^	−3.19 ± 0.49 ^Ha^	1.93 ± 0.11 ^Cb^	1.25 ± 0.10 ^Ba^	1.55 ± 0.09 ^JKa^
AS	0.88 ± 0.06 ^Jab^	−3.79 ± 0.91 ^Ha^	2.85 ± 0.63 ^Aa^	1.74 ± 0.46 ^Aa^	1.49 ± 0.12 ^JKa^
AS-AA	0.78 ± 0.02 ^Jb^	−3.75 ± 0.17 ^Ha^	1.03 ± 0.09 ^Db^	0.79 ± 0.03 ^DEFGb^	0.64 ± 0.02 ^Kc^
AS-VA	0.92 ± 0.03 ^Ja^	−4.87 ± 0.21 ^Ia^	2.14 ± 0.11 ^Cab^	1.14 ± 0.04 ^BCab^	1.04 ± 0.07 ^Kb^
FMS	10.23 ± 0.15 ^Ca^	−0.41 ± 0.01 ^Aa^	1.08 ± 0.00 ^Db^	0.85 ± 0.01 ^DEb^	8.64 ± 0.00 ^Ca^
FMS-AA	3.56 ± 0.14 ^HIb^	−0.85 ± 0.04 ^ABCDb^	1.31 ± 0.02 ^Da^	1.02 ± 0.04 ^BCDa^	3.62 ± 0.25 ^GHb^
FMS-VA	3.91 ± 0.23 ^Hb^	−1.61 ± 0.05 ^EFc^	1.37 ± 0.10 ^Da^	0.92 ± 0.03 ^CDEa^	3.59 ± 0.11 ^GHb^
QS	3.86 ± 0.02 ^Ha^	−3.72 ± 0.38 ^Ha^	1.16 ± 0.02 ^Da^	0.53 ± 0.01 ^Gb^	2.07 ± 0.10 ^IJa^
QS-AA	3.26 ± 0.03 ^Ib^	−4.99 ± 0.16 ^Ib^	1.22 ± 0.07 ^Da^	0.76 ± 0.03 ^DEFGa^	2.37 ± 0.27 ^IJa^
QS-VA	3.78 ± 0.04 ^Hab^	−5.59 ± 0.21 ^Jb^	1.20 ± 0.01 ^Da^	0.72 ± 0.01 ^EFGa^	2.73 ± 0.02 ^HIa^
BS	12.65 ± 0.92 ^Ba^	−0.90 ± 0.05 ^ABCDa^	1.08 ± 0.00 ^Db^	0.77 ± 0.03 ^DEFGb^	9.74 ± 2.28 ^BCa^
BS-AA	7.22 ± 0.16 ^Db^	−1.26 ± 0.11 ^CDEb^	1.11 ± 0.04 ^Db^	0.90 ± 0.10 ^CDEa^	6.49 ± 0.57 ^Dab^
BS-VA	4.67 ± 0.07 ^Gc^	−1.96 ± 0.14 ^FGc^	1.21 ± 0.02 ^Da^	0.92 ± 0.03 ^CDEa^	4.32 ± 0.19 ^FGb^
OS	10.62 ± 0.07 ^Ca^	−0.45 ± 0.01 ^ABa^	1.12 ± 0.04 ^Da^	0.93 ± 0.03 ^CDEa^	9.84 ± 0.2 ^Ba^
OS-AA	6.11 ± 0.08 ^Eb^	−1.42 ± 0.21 ^DEFb^	1.19 ± 0.05 ^Da^	0.89 ± 0.05 ^CDEa^	5.41 ± 0.25 ^DEFb^
OS-VA	5.31 ± 0.27 ^Fc^	−0.63 ± 0.00 ^ABCa^	1.19 ± 0.00 ^Da^	0.95 ± 0.00 ^CDEa^	5.06 ± 0.26 ^EFb^
WMS	ND	ND	ND	ND	ND
WMS-AA	ND	ND	ND	ND	ND
WMS-VA	ND	ND	ND	ND	ND
NMS	18.68 ± 0.10 ^Aa^	−2.54 ± 0.08 ^Gc^	1.06 ± 0.01 ^Dc^	0.57 ± 0.02 ^FGc^	11.04 ± 0.29 ^Aa^
NMS-AA	7.69 ± 0.13 ^Db^	−3.43 ± 0.17 ^Hb^	1.13 ± 0.01 ^Db^	0.81 ± 0.00 ^DEFb^	6.22 ± 0.09 ^DEb^
NMS-VA	4.95 ± 0.06 ^Gc^	−1.09 ± 0.07 ^BCDEa^	1.18 ± 0.02 ^Da^	0.91 ± 0.00 ^CDEa^	4.63 ± 0.10 ^FGc^

Mean ± SD values from triplicate data followed by different lowercase letters of the same origin and different uppercase letters in the same column are significantly different (*p* < 0.05). ND. Texture properties of WMS, WMS-AA, and WMS-VA were not detected.

## Data Availability

The raw data supporting the conclusions of this article will be made available by the authors on request.

## Supplementary Materials

The following supporting information can be downloaded at: https://www.mdpi.com/article/10.3390/foods14132227/s1, Table S1: The 10th (d10), medium (d50), 90th (d90) percentile and span factors of the native and acetylated starch granules; Table S2: Correlation analysis between starch granule size and DS values; Table S3: Correlation analysis between degree of substitution and pasting properties of acetic anhydride modified starches; Table S4: Correlation analysis between degree of substitution and pasting properties of vinyl acetate modified starches.
