# Supplementary material for: Differential Pasting and Rheological Properties of Diverse Underutilized Starches Modified by Acetic Anhydride and Vinyl Acetate
Source: Foods. 2025 Jun 24;14(13):2227. doi: 10.3390/foods14132227 (PMC12249025; doi:10.3390/foods14132227)
Supplement: Supplementary file 1 [file foods-14-02227-s001.zip › foods-3668045-supplementary.pdf]

Table S1. The 10th (d10), medium (d50), 90th (d90) percentile and span factors of the native and acetylated starch granules.

Samples	d10	d50	d90	Span
PMS	5.09±0.01^{Lb}	8.15±0.04^{Jc}	13.05±0.21^{Mc}	0.97±0.02^{JKLc}
PMS-AA	5.28±0.01^{Ka}	8.96±0.05^{Ib}	15.75±0.21^{LMb}	1.17±0.01^{HIJKLb}
PMS-VA	5.24±0.01^{Ka}	9.52±0.08^{Ha}	18.80±0.42^{JKa}	1.43±0.04^{Ha}
AS	1.12±0.01^{Sc}	2.66±0.06^{Nc}	13.10±0.85^{Mc}	4.51±0.23^{Dc}
AS-AA	2.67±0.03^{Oa}	6.67±0.17^{La}	44.75±2.47^{Ca}	6.30±0.20^{Cb}
AS-VA	1.58±0.01^{Rb}	4.82±0.15^{Mb}	36.75±3.18^{Db}	7.29±0.42^{Ba}
FMS	6.20±0.01^{Ic}	10.75±0.07^{Gb}	19.50±0.14^{IJb}	1.24±0.01^{HIJa}
FMS-AA	6.80±0.02^{Ga}	11.65±0.07^{Ea}	20.80±0.14^{GHIJa}	1.20±0.00^{HIJKa}
FMS-VA	6.46±0.03^{Hb}	10.95±0.07^{FGb}	19.85±0.35^{HIJb}	1.22±0.03^{HIJa}
QS	5.02±0.13^{La}	8.32±0.11^{Jb}	44.15±2.05^{Cc}	4.71±0.33^{Dc}
QS-AA	2.58±0.02^{Ob}	7.14±0.18^{Kc}	67.10±2.69^{Bb}	9.04±0.14^{Aa}
QS-VA	2.32±0.04^{Pc}	11.45±0.78^{Ea}	88.15±1.91^{Aa}	7.53±0.34^{Bb}
BS	1.70±0.02^{Qc}	4.48±0.11^{Mc}	13.30±0.85^{Mb}	2.60±0.12^{Ea}
BS-AA	5.20±0.01^{Ka}	8.12±0.01^{Jb}	13.75±0.07^{Mb}	1.06±0.01^{IJKLc}
BS-VA	4.88±0.12^{Mb}	8.39±0.04^{Ja}	16.65±0.49^{KLa}	1.40±0.07^{HIb}
OS	4.63±0.00^{Nc}	10.60±0.00^{Gc}	32.70±0.00^{Eb}	2.64±0.00^{Ea}
OS-AA	5.43±0.01^{Jb}	11.30±0.00^{EFb}	25.85±0.07^{Fc}	1.81±0.00^{Gc}
OS-VA	6.92±0.04^{Fa}	17.30±0.28^{Aa}	45.70±1.98^{Ca}	2.24±0.08^{Fb}
WMS	10.05±0.07^{Bab}	15.15±0.07^{BCab}	22.65±0.07^{GHb}	0.83±0.00^{KLb}
WMS-AA	9.90±0.02^{Cb}	15.05±0.07^{BCDb}	22.65±0.07^{GHb}	0.85±0.00^{KLa}
WMS-VA	10.15±0.07^{Aa}	15.40±0.14^{Ba}	23.10±0.14^{Ga}	0.85±0.00^{KLa}
NMS	9.75±0.06^{Da}	14.95±0.21^{CDa}	22.75±0.35^{GHa}	0.87±0.01^{KLa}
NMS-AA	9.64±0.04^{Ea}	14.65±0.07^{Da}	22.10±0.28^{GHIa}	0.85±0.01^{KLa}
NMS-VA	9.74±0.03^{Da}	14.85±0.07^{CDa}	22.40±0.28^{GHa}	0.85±0.01^{KLa}

Mean ± SD values from triplicate data followed by different lowercase letters of the same origin and different uppercase letters in the same column are significantly different ($p < 0.05$).

Table S2. Correlation analysis between starch granule size and DS values.

	d10	d50	d90	span
Degree of substitution (AA)	-0.07	-0.279	-0.356	0.171
Degree of substitution (VA)	-0.052	0.021	0.512	0.309

Table S3. Correlation analysis between degree of substitution and pasting properties of acetic anhydride modified starches.

	Degree of substitution (AA)
Peak Viscosity	0.478
Breakdown	0.326
Final Viscosity	-0.405
Setback	-0.62

Table S4. Correlation analysis between degree of substitution and pasting properties of vinyl acetate modified starches.

	Degree of substitution (VA)
Peak Viscosity	-0.267
Breakdown	-0.498
Final Viscosity	0.549
Setback	0.319
